# Botanical Report

**Published:** 1811-06

**Authors:** 


### BOTANICAL REPORT.

Four numbers of the Botanist's Repositoty have been published, since we last noticed this work: we shall devote the present Report to a notice of the contents of these.

Anneslea *spinosa*, so called by Dr. Roxburgh in honour of the Right Honourable George Annesley Viscount Valentia, who, we are here told, "*discovered* this plant growing in the Gagra river in Oude, and also about Chittagong." For *discovered* we suppose to understand *saw*, for specimens of this plant were brought by Sir George Staunton, from the province of Kinang, in China, from his return from his embassy to this country, long before Lord Valentia commenced his travels in India. We have not, however, the smallest objection to the name; for, when the Magnates concern themselves at all about natural history, more especially when they undertake laborious travels, with a view of acquiring knowledge therein, we should be sorry to deprive them of an iota of their honorary rewards; and we are pleased to see the most magnificent plants devoted to the record of a fame, so much more meritorious, than that of shining in the annals of the Racing Calendar, or the petty intrigues of a borough election. This is altogether a curious article about Nymphs and Naiades, not more conspicuous for their beauty and elegance, than for their mildness;" and about Anneslea, panther-like, uniting the extremes of beauty and ferocity. We cannot suppose the author is slily insinuating any similarity between his Lordship and his plant, between Annesley and Anneslea. But it gives him an opportunity of introducing something about the armour of vegetables, in which the different kinds are amusingly jumbled together in an unusual figure in rhetoric, an inverted bathos, "*from* the almost imperceptible hair *down* to the lacerating thorn;" and about the browsing of camels, asses, and goats; and about hoping to see the magnificent foliage of Anneslea mantling our ponds. The reasoning, however, on which our author grounds his hopes of our being able to naturalize a tropical production to our climate, is not very convincing. "Have we not," he exultingly exclaims, "already taught the Thea, the Camellia, the Takio, the Moutan, the Yulan, to resist our winters?" Now it unfortunately happens that we have not taught one of these plants to bear cold a jot better than they did ages ago, in the imperial gardens at Pekin. We have indeed had the good sense to discover, that, being natives of climes more rigid than our own, it was not necessary to confine them to our stoves.

We have been so entertained with this article, that we could not withstand the temptation of amusing our readers with parts of it; but we must not forget to say something serious of this very curious kind of water-lily, which has flowered at White Knight's, the seat of the Marquis of Blandford. The flowers according to the figure are but small, in proportion to the immense size of the foliage, sometimes six or eight feet in circumference, the petals are blue, the calyx green on the outside, and red within.

Eugenia *zealanica;* from Boyton, the seat of A. B. Lambert, esq. It is one of the natural order of myrti. A curious observation is here made concerning the germen, which contains sixteen ovula, though the fruit admits of only one seed coming to perfection.

Schinus *dentata;* a native of Owhyhee, and sufficiently hardy to

 thrive thrive well in a sheltered situation in the open ground, and even to produce ripe seeds in favourable seasons, if trained against a wall.

Jussiena *exaltata;* the cattri calambri of the hortus malabaricus, v. 2, p. 97, 150, a new species introduced from the East Indies by Dr. Roxburgh, and communicated to the author by Mr. Lambert, from his seat at Boyton.

Leptospermum *cooparium*, native of New Zealand, and one of the most beautiful of the natural order of the myrti, from that quarter, from the number and duration of its flowers. It was found also by Captain Cook to be very useful, and is the shrub described by him in his second voyage, under the name of the tea-plant.

Ardisia *elegans*; native of Pulo-Pinang, where it grows in moist situations, and by the sides of rivulets, introduced by Mr. Evans, of Stepney, in whose stove it attained the height of nearly five feet. This species appears not to have been before described.

Lotus *australis*. A plant we have before mentioned from the Botanical Magazine.

Barleria *cristata*. Barleria comes very near to justicia; even two of the four stamens are nearly abortive. This plant is likewise from Mr. Evans's collection, as are the three following:

Geodorum *citrinum*, a delicate plant of the family of the orchideæ.

Begonia *evansiana;* said to be discovered by Mr. Evans's collector in the island of Pulo-Pinang. We believe, however, that this plant has been long in the collection at Shew.

Clerodendrum *pyramidale;* supposed to be a new species, also from Pulo-Pinang. Volkameria and Clerodendrum are very unsatisfactorily defined, and several species seem to have been indiscriminately referred to either genus.

Desmanthus *natans* of Wildenow; Mimosa *natans* of Roxburgh's Coromandel plants; Neptunia *natans* of Loureiro. To the character, as here given from Wildenow, the flowering spikes of this plant do not correspond, being neither oblong, nor interrupted, but oval and compact. The roots in Mimosa *natans* have no attachment whatever to the soil, but are produced in fibrous bunches along the stems, which are likewise furnished with a sponge-like substance as it is called, but which must be more of the nature of cork than sponge, for the purpose of preventing the plant from sinking in the water. There is no appearance either of the roots or of this buoyant cork in the figure, nor any mention made of it in the description, nor of its mode of growth. These circumstances leave some doubt in the mind whether the plant here figured be really the Mimosa *natans* of Roxburgh, or the Neptunia *natans* of Loureiro. The specimen from which the drawing was taken, was communicated by Mr. Milne from Mr. Beckford's collection, at Fonthill.

Ardisia *littoralis*, discovered on the shores of Pulo-Pinang by Mr. Evans's collector, and introduced at the same time with Ardisia *elegans* above mentioned. This is probably the same as Ardisia *solanacea* of Roxburgh.

Styrax *officinale*. An old, but still a rare, shrub in our gardens.

Cytisus *elongatus*. The first account we have of this elegant species, is in the rare plants of Hungary, published by Count Waldstein and Dr. Kitabel. Introduced to this country by the indefatigable and skilful curator of the botanic garden at Cambridge.

Liatris *odoratissima.* Introduced by the late Mr. Fraser, of Sloane-square, from North America. This intrepid and zealous traveller has at last sunk under the infirmities induced by his laborious exertions in the acquisition of new plants. Its value consists in the fragrance of the dried foliage, exactly resembling that of the Tonquin bean, and equally durable. Being a native of South Carolina, our summers seem to have too little sun to bring it into flower. The drawing was made from a specimen which bloomed in Mr. Lambert's stove at Boyton. As a flowering plant, it is not superior to our common hemp agrimony, which it somewhat resembles; but if it should thrive well in the open air, and produce its foliage freely, it will prove a very valuable acquisition.

Peliosanthes *humilis*; a diminutive species from Mr. Evans's collection, native of Pinang.

Celosia *cernua*; a new species, introduced from the East Indies by Dr. Roxburgh. It is a very ornamental annual, and may be raised with our Cockscombs and Balsams. To the former it has a near affinity, but is more elegant in its growth.

Ipomæa *insignis.* This most splendid bindweed has been for some years cultivated in the stove of Mr. Benyon, at Englefield, where it extends over the trellis work for about thirty feet, producing numerous bunches of large bell-shaped flowers, of a purplish colour, with a dark centre. Its native country and time of introduction are totally unknown. We have been informed that it was long erroneously supposed to be the West Indian yam.

The second part of the tenth volume of the Transactions of the Linnæan-Society is just published.

